# State of Medical Education in Turkey

**Published:** 1849-03

**Authors:** 


*State of Medical Education in Turkey.*—We are indebted to a medical gentleman, at present residing in Constantinople, for the following details. They point to the existence of a state of matters, of which very imperfect accounts have as yet reached this country; and such as, undoubtedly, is at once the proof and the earnest of a new era in the history of civilization having begun in the East.

"Military hospitals, on a large scale, are either built or a-building in every quarter of the Turkish Empire. There are about one thousand European surgeons attached to the different regiments, two hundred of whom are Jews. The chief professor of the Medical College, Dr. Spitzer, is a Jew. He is also one of the physicians in ordinary to the Sultan. By him I was introduced to H. E. the Hakim Bashy Ismael Effendi, the chief physician of the Ottoman Empire, who kindly permitted me to visit the different lecture-rooms in the Medical College. It is certainly in a very flourishing condition, considering that it has been in existence only eleven years. The pupils are brought, by order of the Sultan, from all departments of the empire, and are lodged, fed, clothed, and educated, at the expense of the government. When qualified as physicians and surgeons, they receive appointments in the army and navy, with salaries of £200 or £300, and upwards, according to rank, without distinction of Jew or Gentile. Until lately, however, there were no Jews in this college; not that the government excluded them, on the contrary, they were invited; but that people, who have been scattered amongst all nations, yet amalgamated with none, would not send their sons to this medical establishment, even although the most flattering prosprects of education and worldly advancement were held out to them. But the government condescended to smooth all the difficulties which stood in the way of the improvement of this section of its subjects. Through its agents, it held interviews on the question of consciencious scruples with the chief Jewish Rabbis: and the result was, not only the guaranteeing liberty of conscience to the Jews who should enter the Medical School, but the assigning to them a distinct portion of the college, so that they might live separate from the Gentiles, the appointment of a superintendent of their own persuasion, to see that their religious duties and services should be strictly observed—also a shocket, or butcher, of their own; and, in short, every arrangement was made to prevent their being constrained to do any thing contrary to their conscience. In the language of last year's report of the college authorities,—'Toutes les difficultés ont été aplanies, et le Gouvernment n'a reculé aucune sacrifice pour exercer aussi son influence civilisatrice sur cette partie des sujets de l'Empire.' The Sultan lately visited the college, and presided at the examination of the candidates for the medical degree. When the pupils are first introduced to the college, they are, for the most part, raw, ignorant, and uncivilized. They are at first taught Turkish, afterwards the Arabic and French languages; next geography, history, arithmetic, and other elementary branches of education, including natural history. They have already a very tolerable museum of natural objects, well preserved and well arranged. A small botanic garden is also attached to the college. After undergoing a thorough elementary education, the pupils enter their medical course, comprising lectures on anatomy, physiology, chemistry, materia medica, practice of physic, surgery, and midwifery. The only room I did not see, was the dissecting-room; it was closed at the time for want of subjects, which it is difficult to procure in a country where so much prejudice against dissection exists, and even against touching a dead body. I was shown into the grand examination room, fitted up with a great throne of state for the Sultan, who presides at the yearly examination of the candidates for the medical degree. There are also a dispensary and hospital attached to the college. The hospital is divided into medical and surgical wards, and a special ward is set apart for diseases of the eye. Dr. Spitzer delivers clinical lectures in the hospital, which he kindly invited me to attend."—*Monthly Jour. Med. Sci.*

CONSTANTINOPLE, March, 1848.

